# Assessment of the safety and gut microbiota modulation ability of an infant formula containing *Bifidobacterium animalis* ssp. *lactis* CP-9 or *Lactobacillus salivarius* AP-32 and the effects of the formula on infant growth outcomes: insights from a four-month clinical study in infants under two months old

**DOI:** 10.1186/s12887-024-05289-7

**Published:** 2024-12-27

**Authors:** Shang-Po Shen, Hung-Chih Lin, Jui-Fen Chen, Hui-Shan Wang, Yen-Yu Huang, Ko-Chiang Hsia, Jia-Hung Lin, Yi-Wei Kuo, Ching-Min Li, Yu-Chieh Hsu, Shin-Yu Tsai, Hsieh-Hsun Ho

**Affiliations:** 1 Division of Neonatology, China Medical University Children’s Hospital, Taichung City, Taiwan; 2 School of Chinese Medicine, China Medical University, Taichung City, Taiwan; 3 Department of Pediatrics, Asia University Hospital, Asia University, Taichung City, Taiwan; 4 Research Product Department, R&D Center, Glac Biotech Co., Ltd, Tainan City, Taiwan; 5 Functional R&D Department, R&D Center, Glac Biotech Co., Ltd, Tainan City, Taiwan

**Keywords:** Probiotics, Infant formula, Gastrointestinal disorders, Infant growth

## Abstract

**Background:**

Breast milk is a natural treasure for infants, and its microbiota contains a rich array of bacterial species. When breastfeeding is not possible, infant formula with probiotics can be used as a sole source or as a breast milk supplement. The main aim of this study was to evaluate the growth outcomes and tolerance of infants consuming an infant formula containing *Bifidobacterium animalis* ssp. *lactis* CP-9 (*B. animalis* CP-9) or *Lactobacillus salivarius* AP-32 (*L. salivarius* AP-32), which were isolated from breast milk and the guts of healthy infants. The safety of these strains in terms of antibiotic resistance and their ability to modulate the gut microbiota were also evaluated.

**Methods:**

One hundred eighty healthy infants were included in this study and separated into three groups: the control group, the *L. salivarius* AP-32 group, and the *B. animalis* CP-9 group. In this clinical study, adverse events, growth effects, and the incidence of allergies and gastrointestinal disorders in infants consuming infant formula containing *B. animalis* CP-9 or *L. salivarius* AP-32 were evaluated. Finally, the impact of the probiotic infant formula on the gut microbiota was elucidated via next-generation sequencing (NGS) analysis.

**Results:**

The 4-month interventional study revealed that body weight, recumbent length, and head circumference were similar among the three groups. No adverse events related to the intervention were observed. The microbiota composition was more diverse on day 0 and became more uniform by month 4. *B. animalis* CP-9 and *L. salivarius* AP-32 were found to be susceptible to streptomycin, tetracycline, erythromycin, clindamycin, chloramphenicol, and ampicillin.

**Conclusions:**

The use of infant formula containing *B. animalis* CP-9 and *L. salivarius* AP-32 was considered safe and well tolerated, with no adverse events observed during the study. While these strains showed low antibiotic resistance and no immediate concerns related to antibiotic resistance genes, further research is needed to comprehensively assess their long-term safety and efficacy and the potential risk of horizontal gene transfer in broader contexts.

**Trial registration:**

The trial was registered with the US Library of Medicine (clinicaltrials.gov) with the number NCT03993301 on 20/06/2019.

**Supplementary Information:**

The online version contains supplementary material available at 10.1186/s12887-024-05289-7.

## Introduction

Over the first year of life, nutrition is the principal regulator of growth, with a minimal contribution from growth hormone. Breastfeeding is a natural and preferred method of feeding [1]. When breastfeeding is not possible, formula can be used as the sole nutrient source or as a supplement to breast milk. Infant formula containing probiotics is commonly used worldwide. A major need when addressing the issue of feeding healthy infants with infant formula is the safety and tolerance of the formula when it is administered for extended periods. Concerns related to the early administration of infant formula containing probiotics include the timing, duration, and delivery in the form of a specific matrix (infant formula), which could be the only source of nutrients for an infant over a prolonged period [2]. Some cases of human infection caused by lactic acid bacteria have been reported, raising public concerns about food safety [3]. Antibiotic resistance testing of probiotics is advised to ensure that antibiotic resistance determinants are not introduced into a context where these genes are at risk of being transferred to pathogenic organisms [4].

In 2010, the Committee of Nutrition of the European Society for Paediatric Gastroenterology, Hepatology and Nutrition (ESPGHAN) commented on infant formula containing probiotics (and/or prebiotics) [5]. Probiotics are defined as ‘‘live microorganisms that, when administered in adequate amounts, confer a health benefit on the host” [6]. As one of the earliest identified probiotic strains, *Lactobacillus rhamnosus* GG ATCC 53103 (LGG) has been shown to have a positive effect on the immune system and gastrointestinal health of children [7–9]. Even though no adverse effects have been reported thus far, a longitudinal study is necessary to evaluate the effects of probiotic-enriched formula on growth and the gut microflora in healthy term infants [10]. Growth studies should include at least measurements of weight, length, and head circumference [11]. Allergic diseases and gastrointestinal illnesses are common health problems in infants, despite their growth. Gastrointestinal illnesses are a major cause of morbidity and mortality in children in developing countries [12, 13]. Allergic diseases are becoming increasingly common worldwide and result from complex interactions between genetic susceptibility and environmental factors [14, 15]. Because allergic sensitization may occur as early as gestation through the first 6 months of life, intervention strategies, such as probiotic administration, are needed for this critical period [16].

*Bifidobacterium animalis* ssp. *lactis* CP-9 was separated from the breast milk, and *Lactobacillus salivarius* AP-32 was separated from the healthy human gut. Both strains were analysed via whole-genome sequencing, and several studies were performed to examine their beneficial effects on human health. The safety of *B. animalis* CP-9 has been assessed in rodents [17], and the combination of *B. animalis* CP-9 with other probiotic strains has been shown to have antioxidative effects in middle-aged mice [18]. *L. salivarius* AP-32 exhibited antibacterial activity against *Helicobacter pylori* and reduced lymphocyte infiltration and inflammatory chemokine expression in *H. pylori-*infected rats [19]. In addition, *L. salivarius* AP-32 ameliorated mitochondrial function and reshaped the gut microbiota in rats with Parkinson’s disease [20, 21]. Both *B. animalis* CP-9 and *L. salivarius* AP-32 exhibited antibacterial activity against oral pathogens and increased IgA concentrations in the oral mucosa [22, 23]. Multistrain probiotic supplements containing *B. animalis* CP-9 and *L. salivarius* AP-32 reduced inflammation and attenuated glycaemic levels in rats with type 2 diabetes and patients with type 1 diabetes [24, 25]. Moreover, the combination of *B. animalis* CP-9, *L. salivarius* AP-32, and *L. rhamnosus* bv-77 improved the blood lipid profile in obese rats [26] and ameliorated obesity-related gut dysbiosis in obese children [27]. Although *B. animalis* CP-9 and *L. salivarius* AP-32 were manufactured for human use more than one decade ago, their safe use in infant formula in particular still needs to be evaluated.

The main aim of this study was to evaluate the growth outcomes of infants consuming infant formula containing *B. animalis* CP-9 or *L. salivarius* AP-32. The safety of *B. animalis* CP-9 and *L. salivarius* AP-32 use was also investigated with respect to antibiotic resistance.

## Materials and methods

### Clinical study in infants

#### Inclusion and exclusion criteria

This double-blind randomized trial was performed at China Medical University Children's Hospital. All the infants were divided into three groups: (I) the probiotic A formula (*L. salivarius* AP-32, 10^7^ colony-forming units (CFU)/g) group and (II) the probiotic B formula (*B. animalis* CP-9, 10^7^ CFU/g) group. The control group received (III) regular formula (Youluck infant formula). The probiotic lyophilized powder was sponsored by Glac Biotech Co., Ltd. (Tainan, Taiwan), and the infant formula was manufactured by Youluck International, Inc., Taiwan. This study adhered to the guidelines of the Declaration of Helsinki, was approved by the China Medical University & Hospital Research Ethics Committee (IRB No. CMUH108-REC2-005), and was registered with the US Library of Medicine (www.clinicaltrial.gov) with the identifier NCT03993301. Infant growth and incidence of allergies and gastrointestinal disorders in infants were assessed in this study. Written informed consent was obtained from the parents or legal guardians of all participants, and only those meeting the inclusion and exclusion criteria detailed below were enrolled in the study.

The inclusion criteria for infants were as follows: (1) healthy infants who were born at term (≥ 37 weeks) via vaginal delivery, with birth weights greater than 2,500 g; and (2) infants who were expected to breastfeed. Infants were eligible for the trial after informed consent was signed by the parents. The exclusion criteria for infants were as follows: the presence of chronic diarrhoea; a diagnosis of haemangioma, cerebral haemorrhage, severe asphyxia (stage III), foetal chromosomal abnormalities, cyanotic congenital heart disease, intestinal hypoplasia or abnormal immune function after birth, liver failure, allergies, gastroenteritis, or respiratory infection; breastfeeding within two months after birth; use of other probiotic products within two weeks after birth; or treatment with antibiotics during an acute infection.

### Study populations

The purpose of this trial was to evaluate the effect of infant formula containing probiotics on neonatal growth. The main endpoint was weight gain after four months of intervention. To examine whether the weight gain of the experimental group was different from that of the control group (placebo), the sample size was calculated according to the method of Puccio et al. in 2007 [28]. In that study, the average weight gain rate increased by 3.9 ± 6.1 g/day in babies fed infant formula containing probiotics compared with that in babies fed infant formula without probiotics. Forty-five evaluable infants per group were needed to have 85% power of the test at the significance level of α = 0.05. To accommodate the expected 25% dropout rate, a total of 60 infants per group were enrolled in the study. The physiological values were evaluated in the per-protocol population (per-protocol analysis).

### Intervention

The infant formula with probiotics (10^7^ CFU/g) or the regular formula were prepared by nurses who were blinded to the groupings. The team members followed the orders from a sealed envelope. Therefore, the only personnel who were aware of the infants’ group assignments were the investigators. The study involved a 4-month probiotic supplementation period, during which it was requested that the infants consume at least 2 scoops (17.2 g) of infant formula per day. The formula was dissolved in warm water (≤ 37 °C) before feeding. All infants were monitored for adverse conditions such as vomiting, diarrhoea or bloating.

### In vitro safety assessment

#### Minimum inhibitory concentration (MIC)

Single colonies of *B. animalis* CP-9 and *L. salivarius* AP-32 were prepared from their stock cultures via the quadrant streaking method. The determination of the MICs of various antibiotics against *B. animalis* CP-9 and *L. salivarius* AP-32 was conducted by the Food Industry Research and Development Institute (FIRDI) in Hsinchu City, Taiwan. For this determination, the microdilution method outlined in the ISO 10932 guidelines (ISO 10932: 2012, 'Milk and milk products—Determination of the minimal inhibitory concentration of antibiotics applicable to Bifidobacteria and no enterococcal lactic acid bacteria') was followed. The data were aligned with FIRDI Report Nos. 110SN00745 and 110SN01622.

### Genome annotation

The whole-genome sequences of *B. animalis* CP-9 and *L. salivarius* AP-32 were obtained by submitting samples to the BGI Genomics Sequencing Service (Guangdong, China). After genome sequence assembly, the drug resistance-related genes and mobile element information were annotated with the Rapid Annotation Subsystem Technology server (http://RAST.nmpdr.org/, version 2.0) [29]. Moreover, promoter analysis was performed with the online tool SAPPHIRE (https://sapphire.biw.kuleuven.be/) to predict whether there was a promoter sequence structure upstream of the drug resistance-related genes. The similarity of the sequences is indicated by the E value.

### Faecal DNA extraction and next-generation sequencing (NGS) analysis

Faecal samples were collected from all the recruited infants 2 times (day 0 and month 4 of the study). Bacterial DNA was extracted from faecal samples with the QIAamp Fast DNA Stool Mini Kit (Qiagen, Germany), with some modifications. Briefly, the storage buffer was removed from the stool sample by centrifugation at 13,200 rpm for 10 min, after which the cells were lysed with InhibitEX buffer. After homogenization, the processed supernatant was obtained by adding proteinase K and ethanol. Finally, a QIAamp spin column was used to obtain the DNA from the supernatant, and the DNA was eluted with elution buffer. The DNA concentration and purity were monitored with a NanoDrop 2000 spectrophotometer (Thermo Fisher Scientific, United States), and a 10X dilution was then performed with elution buffer. The standard V3–V4 region of the 16S rRNA gene was used to construct the gut microbiome library. DNA amplification was carried out with KAPA HiFi Hotstart Readymix (Roche, United States), and purification was performed with AMPure XP magnetic beads (Beckman Coulter, United States). The quality and quantity of the library were assessed by a Fragment Analyser (Advanced Analytical, United States) and a Qubit 3.0 Fluorometer, respectively. Afterwards, the library sequence was analysed with MiSeq (Illumina, United States) with paired-end reads (2 × 301 nt) and at least 100,000 reads per sample.

The quality control and feature table construction of the resulting sequence data were performed with QIIME 2 version 2020.11 (https://qiime2.org/, accessed on 23 Aug 2023) in conjunction with the DADA2 pipeline. Quality filtering, denoising, and chimaera removal were performed to create amplicon sequence variants (ASVs) for subsequent in-depth analyses. The Shannon index was used to measure alpha diversity. Beta diversity was examined with the unweighted UniFrac, a metric calculated via MicrobiomeAnalyst (https://www.microbiomeanalyst.ca/, accessed on 29 Aug 2023). Permutational multivariate analysis of variance (PERMANOVA) was applied to examine and validate the significant differences in beta diversity. The Greengenes (v13.8) 99% operational taxonomic units were used as reference sequences to assign ASVs. The data are presented as the means ± standard deviations (SDs), and significant differences were determined with Student’s t test. The *p* values were adjusted via the false discovery rate (FDR) with Benjamini‒Hochberg (BH) correction to obtain adjusted *p* values (*q* values). A *q* value less than 0.05 was considered to indicate statistical significance.

### Statistical analysis

All the data are presented as the means ± SDs. Pearson's chi-square test was used for categorical variables, and the Kruskal‒Wallis test was used for continuous variables. All the statistical analyses were performed via SPSS 19.0 (IBM Co., United States), and the significance level was set at *p* < 0.05.

## Results

### Disposition of subjects

**Fig. 1:**
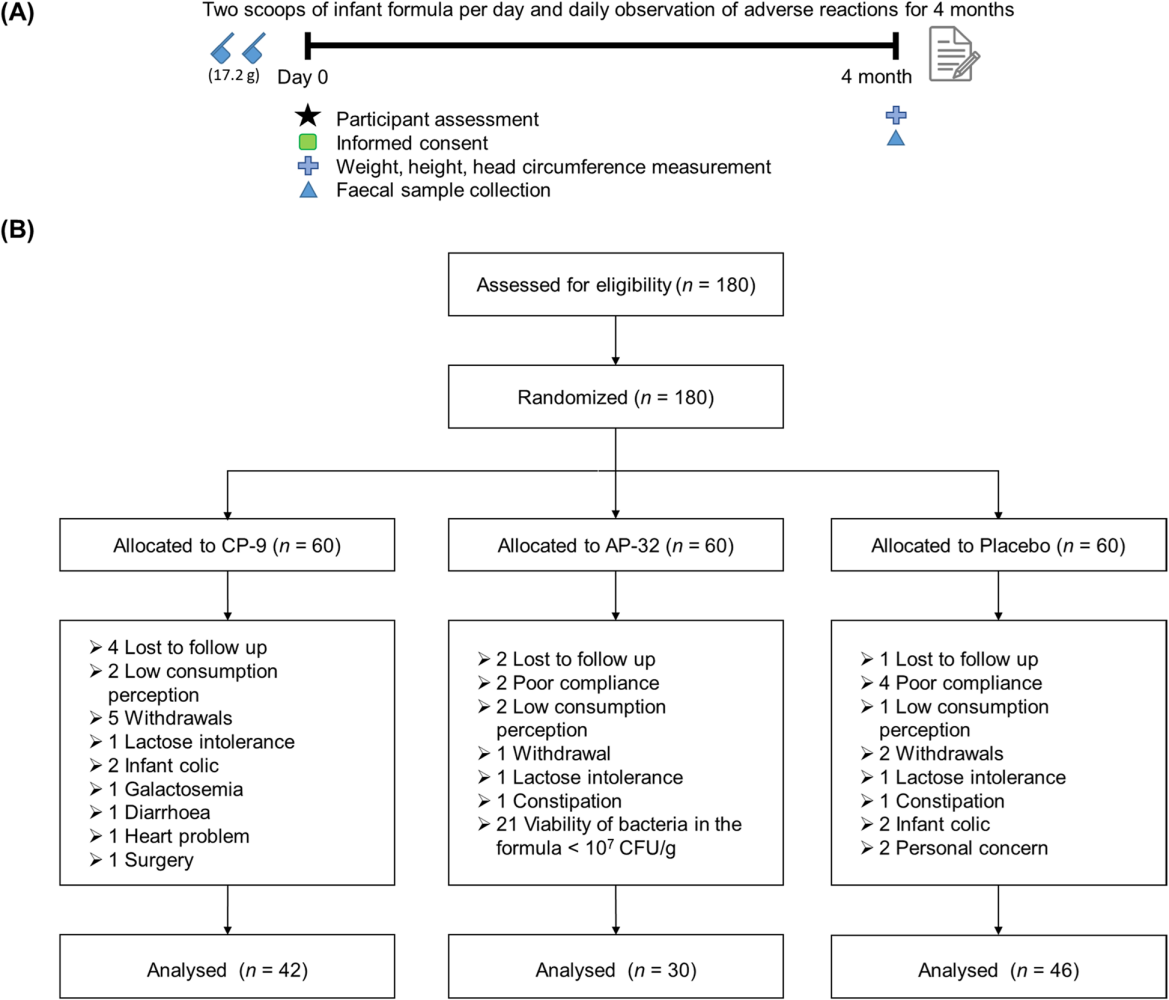
Flow chart of the study design and subjects. **A** It was requested that the study infants consume at least 2 scoops (17.2 g) of infant formula per day for 4 months. **B** One hundred eight (180) healthy, full-term infants were enrolled and randomized to the CP-9 (*n* = 60), AP-32 (*n* = 60), or placebo (*n* = 60) groups. All randomized subjects received allocated treatment. One hundred eighteen (118) subjects completed the 4-month treatment

The planned enrolment was 180 evaluable subjects. The study ended in August 2022 because of the shelf-life of the infant formula. From February 2020 through August 2022, one hundred eighty (180) healthy, full-term, spontaneous vaginally delivered infants were enrolled and randomized to the CP-9 (*n* = 60), AP-32 (*n* = 60), or placebo (*n* = 60) groups. All randomized subjects received the allocated treatment. The study involved a 4-month probiotic supplementation period, during which it was requested that infants consume at least 2 scoops (17.2 g) of infant formula per day (Fig. 1A). One hundred eighteen (118) infants completed the 4-month treatment, the parents of eight infants (4.4%) withdrew consent, and seven infants (3.9%) were lost to follow-up before completing the treatment (Fig. 1B). The dropout rate in the CP-9 group was 30% for reasons including low consumption perception, infant colic, lactose intolerance, galactosemia, diarrhoea, heart problems, and surgery. The dropout rate in the AP-32 group was 50.0%, mainly because of factors such as the low viability of bacteria in the formula (< 10^7^ CFU/g), along with isolated cases of poor compliance, low consumption perception, lactose intolerance, and constipation. The dropout rate in the placebo group was 23.3% for reasons including poor compliance, infant colic, personal concern, low consumption perception, lactose intolerance, and constipation.

### Subject demographics and characteristics

Subject demographics and baseline characteristics are summarized in Table 1. The percentage of females was slightly greater in the AP-32 group (63.3%), and the percentage of males was slightly greater in the placebo group (56.5%). The sex difference was not significant between the probiotic group and the placebo group (*p* = 0.237). The gestational ages were 38.59 ± 0.91 weeks, 38.67 ± 0.87 weeks, and 38.33 ± 0.88 weeks in the placebo, CP-9, and AP-32 groups, respectively (*p* = 0.291). The baseline ages were 2.63 ± 4.52 days, 4.10 ± 9.20 days, and 3.07 ± 6.06 days in the placebo, CP-9, and AP-32 groups, respectively (*p* = 0.410). The treatment durations were 132.80 ± 10.59 days, 131.07 ± 9.96 days, and 131.70 ± 8.81 days in the placebo, CP-9, and AP-32 groups, respectively (*p* = 0.696). The ages when the subjects finished treatment at month 4 were 134.43 ± 11.86 days, 134.17 ± 14.21 days, and 133.77 ± 10.07 days in the placebo, CP-9, and AP-32 groups, respectively (*p* = 0.826). Taken together, the data indicate that the demographic and baseline characteristics were similar among the treatment groups.

**Table 1:** Subject demographics and characteristics

	Placebo	CP-9	AP-32	*p* value
Sex (M/F)	26/20 (56.5%/43.5%)	20/22 (47.6%/52.4%)	11/19 (36.7%/63.3%)	0.237
Gestational age (weeks)	38.59 ± 0.91	38.67 ± 0.87	38.33 ± 0.88	0.291
Baseline age (days)	2.63 ± 4.52	4.10 ± 9.20	3.07 ± 6.06	0.410
Duration (days)	132.80 ± 10.59	131.07 ± 9.96	131.70 ± 8.81	0.696
Age at month 4 (days)	134.43 ± 11.86	134.17 ± 14.21	133.77 ± 10.07	0.826

The data are presented as the number and percentage of subjects (n, %) or mean ± standard deviations (SDs)

*M* Male, *F* Female

### The probiotic formula did not affect the growth of the infants

**Fig. 2:**
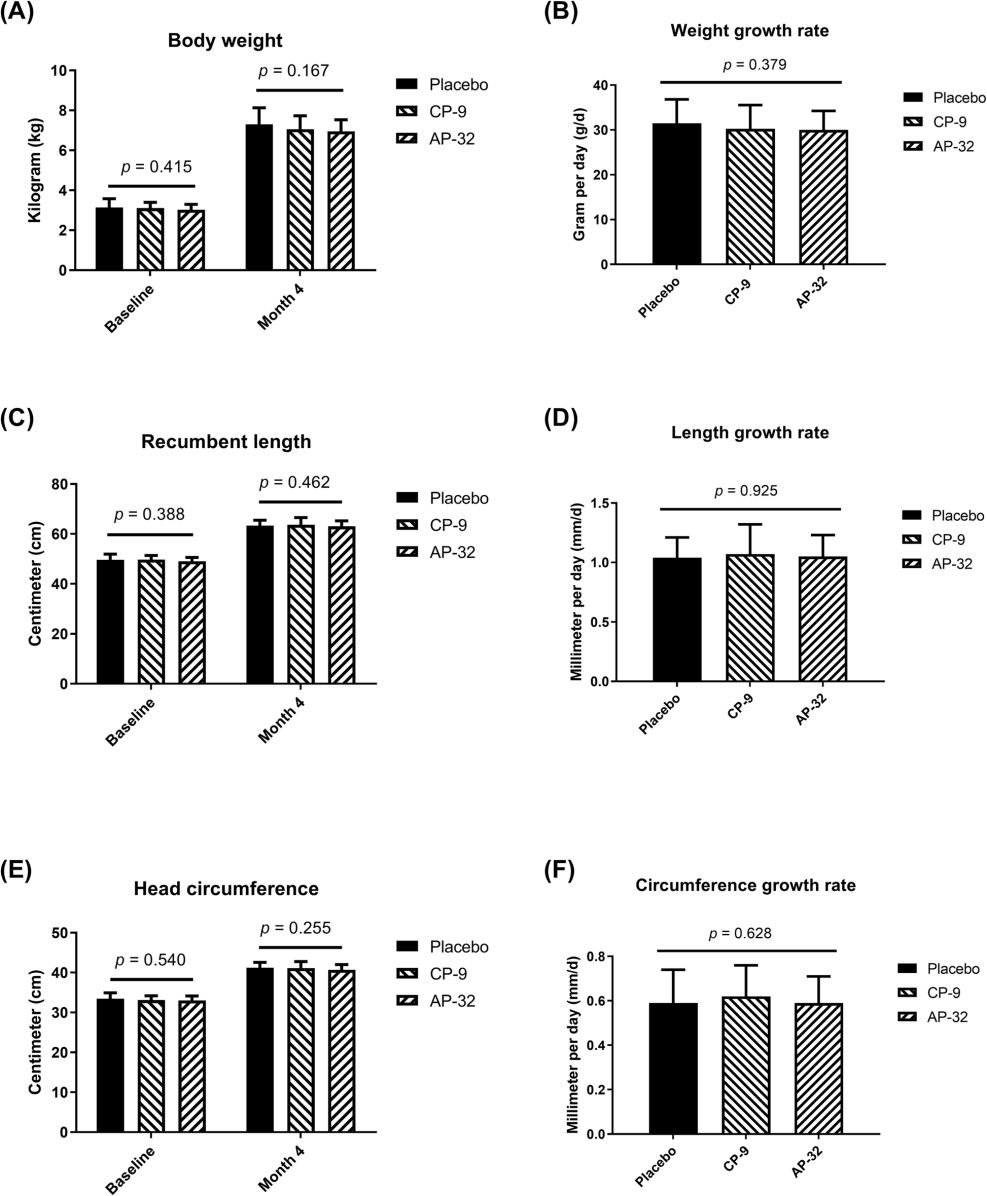
Body weight, recumbent length, and head circumference were measured. The (**A**) body weight, (**C**) recumbent length, and (**E**) head circumference were measured on day 0 and month 4. Growth was converted to the daily rate of (**B**) body weight gain, (**D**) recumbent length gain, and (**F**) head circumference gain

One of the endpoints was the weight gain rate (in grams/day) of the individual infants during the 4 months of the study. The baseline weights recorded on day 0, before the intervention started, were similar among all groups (placebo: 3.14 ± 0.43 kg; CP-9: 3.10 ± 0.29 kg; AP-32: 3.02 ± 0.28 kg; *p* = 0.415; Fig. 2A), and the body weights measured at month 4 of the intervention were not significantly different (placebo: 7.30 ± 0.83 kg; CP-9: 7.05 ± 0.68 kg; AP-32: 6.95 ± 0.58 kg; *p* = 0.167; Fig. 2A). The weight gain rate was calculated by subtracting the baseline weight from the weight at the end of the study (month 4), which was then divided by the treatment duration. The mean weight gain was similar between the two probiotic groups and the placebo group (placebo: 31.47 ± 5.35 g/day; CP-9: 30.24 ± 5.30 g/day; AP-32: 30.03 ± 4.23 g/day; *p* = 0.379; Fig. 2B).

The growth rates (in millimetres/day) of recumbent length and head circumference were two other endpoints of the assessment. The baseline length and circumference recorded on day 0, before the intervention started, were similar among all groups (placebo: 49.62 ± 2.26 cm; CP-9: 49.68 ± 1.68 cm; AP-32: 49.04 ± 1.51 cm; *p* = 0.388; placebo: 33.45 ± 1.48 cm; CP-9: 33.11 ± 1.06 cm; AP-32: 33.03 ± 1.13 cm; *p* = 0.540; Fig. 2C and E); the length and circumference measured at month 4 of the intervention were not different (placebo: 63.33 ± 2.17 cm; CP-9: 63.67 ± 2.91 cm; AP-32: 63.01 ± 2.19 cm; *p* = 0.462; placebo: 41.21 ± 1.36 cm; CP-9: 41.13 ± 1.63 cm; AP-32: 40.7 ± 1.30 cm; *p* = 0.255; Fig. 2 C and E). The mean growth rates in terms of length and circumference were similar between the two probiotic groups and the placebo group (*p* = 0.925 and *p* = 0.628; Fig. 2D and F).

### No intervention-related adverse events were observed by the paediatricians

During the study, forty-six (39.0%) of the 118 infants experienced one adverse event (AE): 16 infants (38.1%) in the CP-9 group, 16 infants (53.3%) in the AP-32 group, and 14 infants (30.4%) in the placebo group (Table 2). The gastrointestinal system was the most common organ system involved (*n* = 24, 20.3%), with infantile colic being the most common manifestation (*n* = 15, 12.7%). A few cases of abdominal bloating (*n* = 5, 4.2%) were recorded, while cases of constipation, enteritis, anal fissures, and inguinal hernia were all single cases (*n* = 1, 0.8%). There was no apparent difference between the probiotic groups and the placebo group in terms of the number of infants with these AEs (*n* = 8/9/7, *p* = 0.284). The integumentary system was the second most common organ system involved, with 22 (18.6%) infants experiencing AEs within this category. Atopic dermatitis (*n* = 11, 9.3%) was the most common skin AE, followed by seborrheic dermatitis (*n* = 8, 6.8%), rash (*n* = 2, 1.7%), and infantile eczema (*n* = 1, 0.8%). The number of patients with these AEs was similar between the two probiotic groups and the placebo group (*n* = 8/7/7, *p* = 0.672). A total of 46 AEs were reported during the study, and none of them were diagnosed as being possibly related to the study product by paediatricians.

**Table 2:** Summary of gastrointestinal disorders and allergic diseases

	Total (*n* = 118)	Placebo (*n* = 46)	CP-9 (*n* = 42)	AP-32 (*n* = 30)	*p* value
**Total**	**46 (39.0%)**	**14 (30.4%)**	**16 (38.1%)**	**16 (53.3%)**	**0.134**
**Gastrointestinal disorders**	**24 (20.3%)**	**7 (15.2%)**	**8 (19.0%)**	**9 (30.0%)**	**0.284**
Constipation	1 (0.8%)	–	1 (2.4%)	–	0.402
Infantile colic	15 (12.7%)	5 (10.9%)	5 (11.9%)	5 (16.7%)	0.745
Abdominal bloating	5 (4.2%)	2 (4.4%)	1 (2.4%)	2 (6.7%)	0.672
Enteritis	1 (0.8%)	–	–	1 (3.3%)	0.228
Anal Fissures	1 (0.8%)	–	–	1 (3.3%)	0.228
Inguinal hernia	1 (0.8%)	–	1 (2.4%)	–	0.402
**Allergic diseases**	**22 (18.6%)**	**7 (15.2%)**	**8 (19.0%)**	**7 (23.3%)**	**0.672**
Seborrheic dermatitis	8 (6.8%)	1 (2.2%)	4 (9.5%)	3 (10.0%)	0.281
Atopic dermatitis	11 (9.3%)	5 (10.9%)	3 (7.1%)	3 (10.0%)	0.826
Rash	2 (1.7%)	1 (2.2%)	–	1 (.3.3%)	0.530
Infantile eczema	1 (0.8%)	–	1 (2.4%)	–	0.402

### *B. animalis *CP-9 and* L. salivarius* AP-32 were susceptible to most of the antibiotics listed by the European Food Safety Agency (EFSA)

The antimicrobial resistance patterns of *B. animalis* CP-9 and *L. salivarius* AP-32 were assessed by comparing the observed MICs with the most recent EFSA breakpoint values (Table 3). *B. animalis* CP-9 was susceptible to vancomycin, streptomycin, tetracycline, erythromycin, clindamycin, chloramphenicol, and ampicillin, with MIC values below the cut-off established by the EFSA. However, *B. animalis* CP-9 displayed an MIC of 128 μg/mL for gentamicin. On the other hand, *L. salivarius* AP-32 was susceptible to gentamicin, streptomycin, tetracycline, erythromycin, clindamycin, chloramphenicol, and ampicillin, with MIC values less than the EFSA cut-off. Nevertheless, *L. salivarius* AP-32 displayed an MIC of 256 μg/mL for kanamycin. These data suggest that *B. animalis* CP-9 and *L. salivarius* AP-32 might be intrinsically resistant to gentamicin and kanamycin, respectively.

**Table 3:** Antibiotic susceptibility profiles (reported as MICs (μg/mL)) obtained via the microdilution method in accordance with ISO 10932 guidelines^a^

Antibiotic	CP-9	AP-32
Gentamicin	128/64	8/16
Vancomycin	1/2	n.r.^b^
Streptomycin	64/128	64/64
Tetracycline	4/8	4/8
Erythromycin	0.25/1	0.5/1
Clindamycin	0.25/1	0.5/1
Chloramphenicol	4/4	4/4
Ampicillin	2/2	0.5/4
Kanamycin	n.r.^b^	256/64

*MICs* Minimum inhibitory concentrations

^a^The results are presented as the measured value/cut-off value in EFSA Journal 2012; 10(6):2740

^b^*nr* not reported

**Fig. 3:**
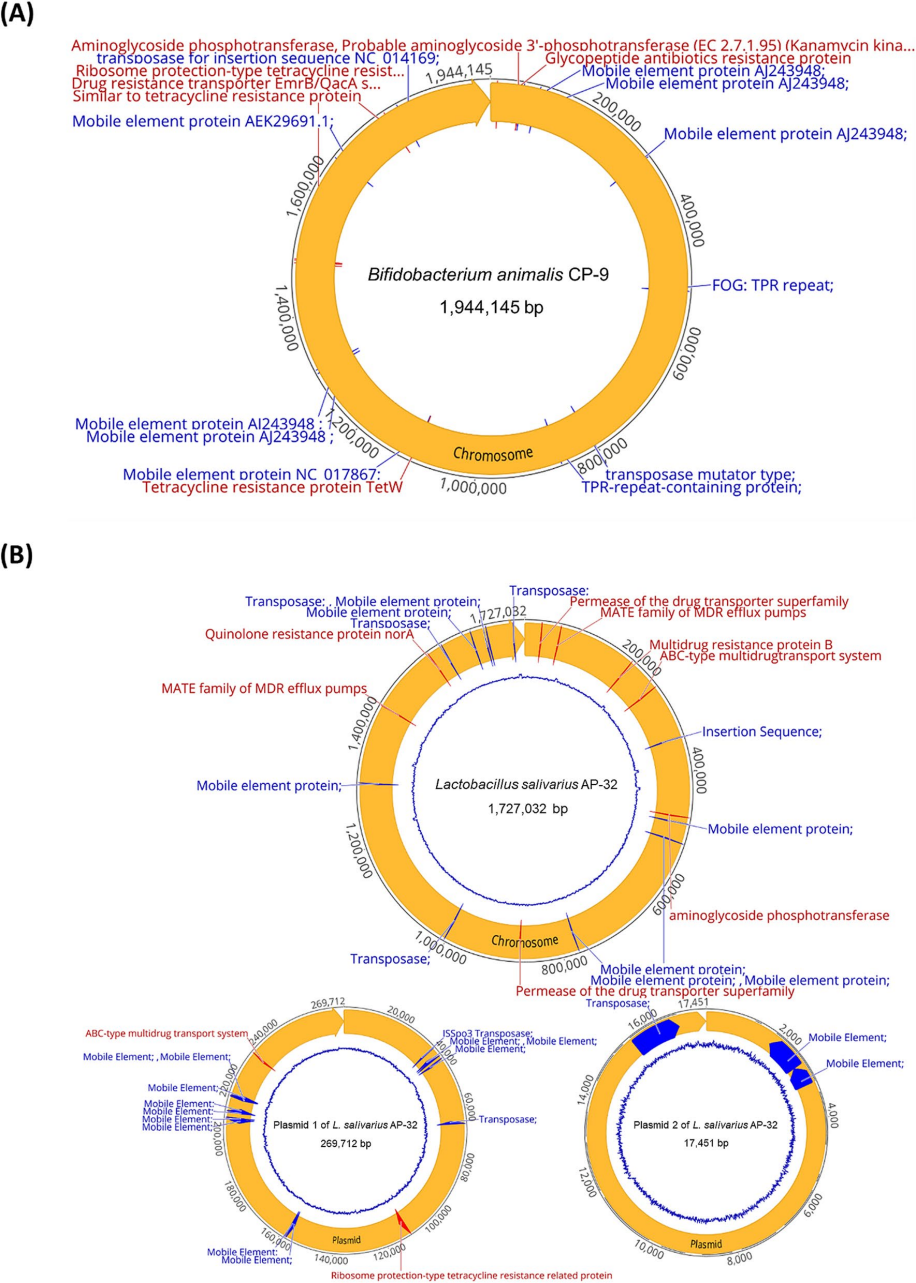
Distribution of antibiotic resistance genes and mobile element genes. **A** *B. animalis* CP-9 contained only one circular chromosome (1,944,145 bp) and no plasmid. **B** *L. salivarius* AP-32 contained one circular chromosome (1,727,032 bp) and two plasmids (269,712 bp and 17,451 bp). Drug resistance genes are indicated in red, and mobile element genes are indicated in blue

To understand the nature of gentamicin and kanamycin resistance in *B. animalis* CP-9 and *L. salivarius* AP-32, respectively, gene annotation of the genome sequences was performed. Gentamicin belongs to the aminoglycoside class of antibiotics. The genome of *B. animalis* CP-9 contained only one circular chromosome (1,944,145 bp) and no plasmid (Fig. 3A). These results suggest that a potential gene structure may contribute to the increased gentamicin resistance of *B. animalis* CP-9 (Table S1). Considering that a potential gene structure was not near any mobile elements or transposases, this resistance is likely intrinsic. Kanamycin is also an aminoglycoside. The genome of *L. salivarius* AP-32 consists of one circular chromosome (1,727,032 bp) and two plasmids (269,712 bp and 17,451 bp; Fig. 3B). The gene annotation indicated a potential gene structure that was not near any mobile elements or transposases, suggesting that this resistance could be intrinsic (Table S2).

### The diversity of the gut microbiota changed with age

**Fig. 4:**
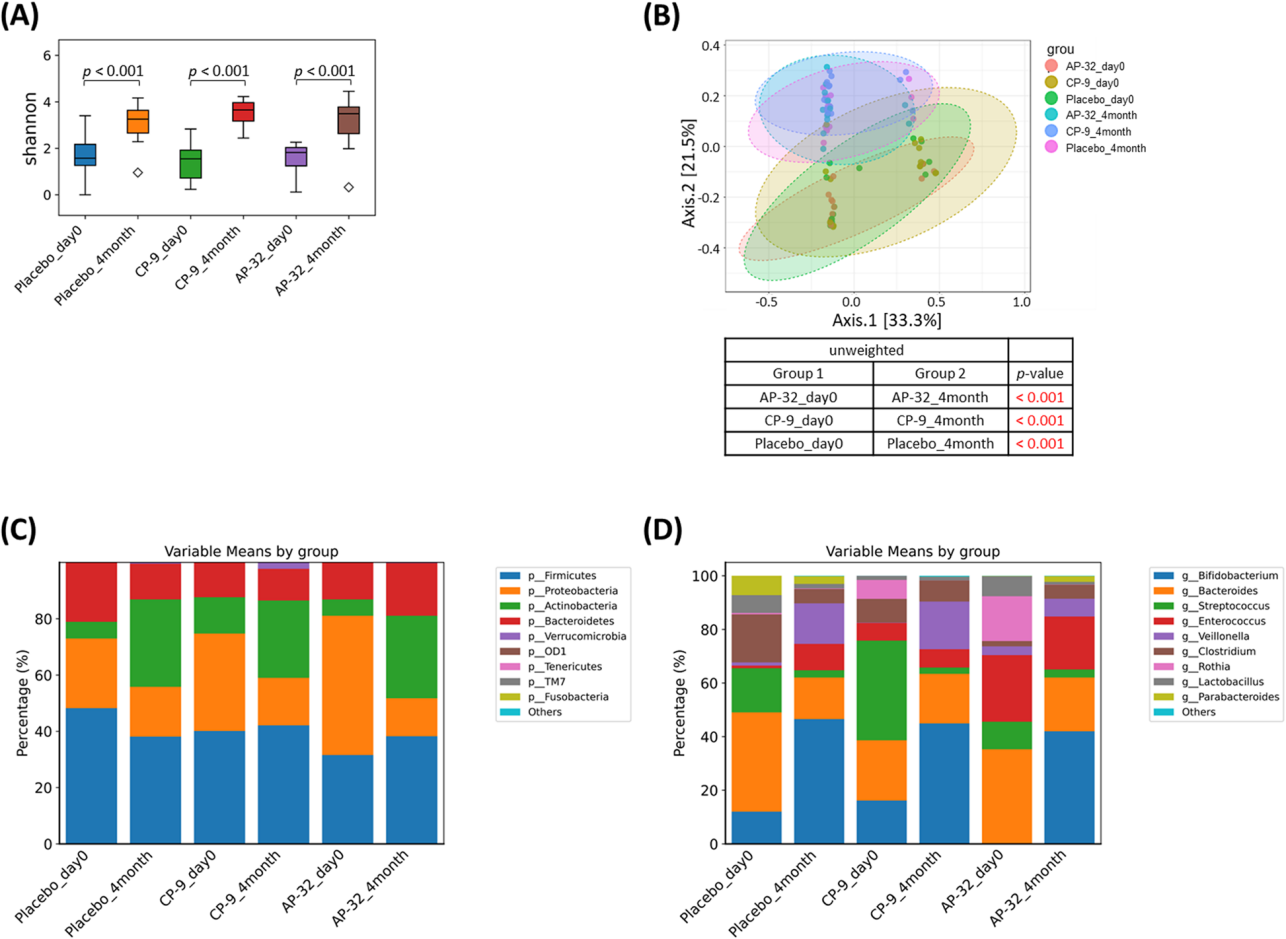
Modulation of the microbiota in faecal samples collected on day 0 and month 4. **A** Alpha diversity, (**B**) beta diversity, (**C**) proportions of the 10 most abundant phyla, and (**D**) proportions of the 10 most abundant genera

**Fig. 5:**
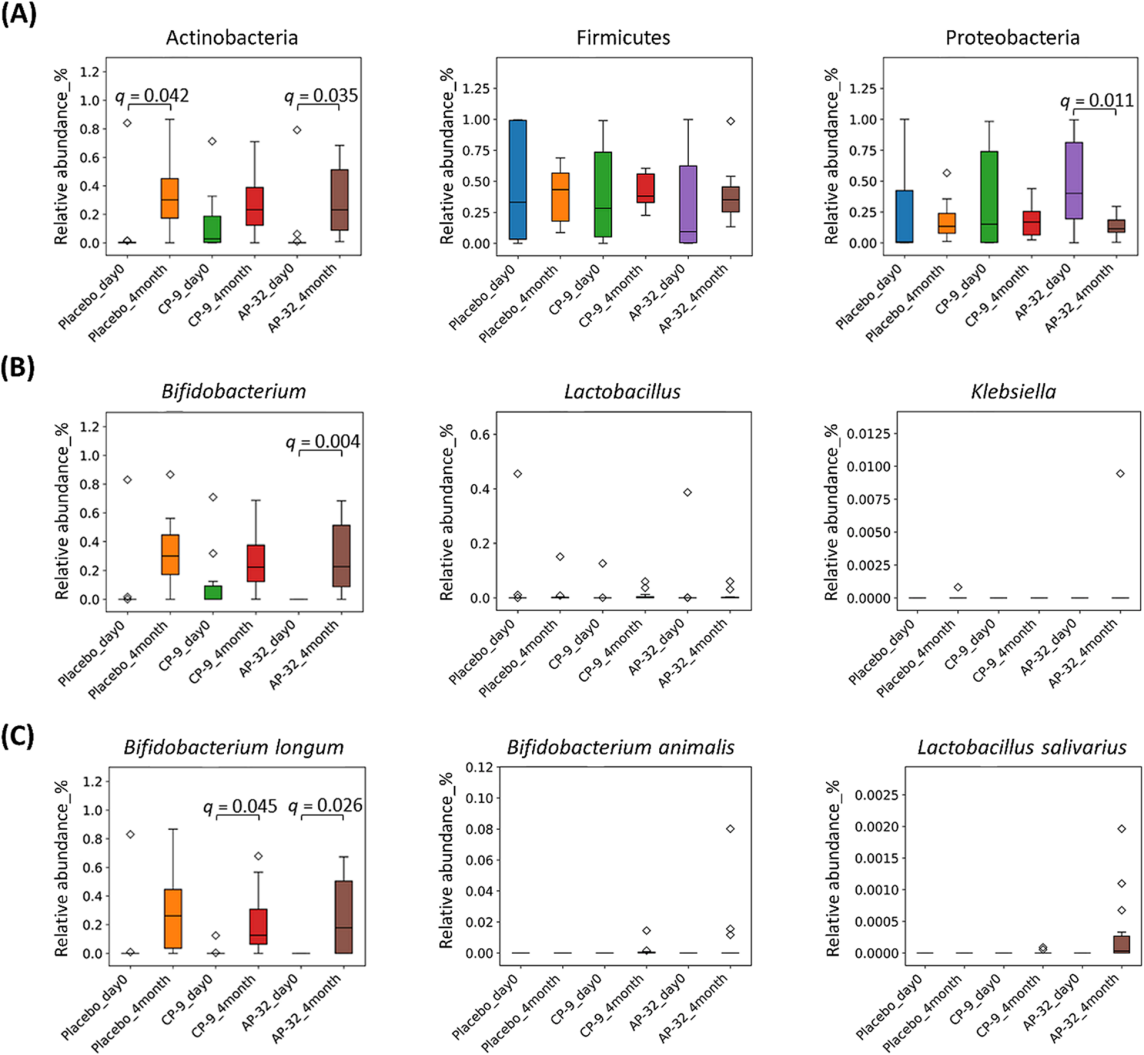
The genera and species involved in the modulation of the microbiota in faecal samples collected on day 0 and at month 4. **A** Phyla: Actinobacteria, Firmicutes, and Proteobacteria; (**B**) genera: *Bifidobacterium*, *Lactobacillus*, and *Klebsiella*; (**C**) species: *Bifidobacterium longum*, *Bifidobacterium animalis*, and *Lactobacillus salivarius*. The *q* value was the *p* value adjusted using the false discovery rate (FDR) with Benjamini‒Hochberg (BH) correction

In all the groups, the alpha diversity increased, and the beta diversity shifted at month 4 (*p* < 0.001; Fig. 4A and B). There were no significant differences in either alpha or beta diversity among the three groups on day 0 or at month 4. The four most abundant phyla in all the groups were Firmicutes, Proteobacteria, Actinobacteria, and Bacteroidetes. The proportions of Firmicutes and Bacteroidetes remained similar between day 0 and month 4. Interestingly, at month 4, the percentage of Proteobacteria decreased, whereas that of Actinobacteria increased (Figs. 4C and 5A). The ten most abundant genera were *Bifidobacterium, Bacteroides, Streptococcus, Enterococcus, Veillonella, Clostridium, Rothia, Lactobacillus,* and *Parabacteroides*, among others. The proportions of the top 10 genera varied among the three groups on day 0 and became more uniform by month 4 (Fig. 4D).

The abundance of the *Bifidobacterium* genus increased in correlation with the increase in the Actinobacteria phylum in all three groups (Fig. 5A and B). Although the content of the *Lactobacillus* genus remained unaffected by the intervention, the proportions of the *Streptococcus, Enterococcus, and Veillonella* genera within the Firmicutes phylum were altered (Figure S1). Notably, the abundance of *Bifidobacterium longum* significantly increased in the CP-9 and AP-32 groups, whereas supplementation with *B. animalis* CP-9 did not lead to a greater proportion of *B. animalis* species (Fig. 5C). Similarly, supplementation with *L. salivarius* AP-32 did not increase the proportion of *L. salivarius* species (Fig. 5C). No significant alterations were identified in either *Klebsiella* (Fig. 5B) or other genera (Figure S2), despite the concurrent reduction in the abundance of the Proteobacteria phylum (Fig. 5A).

## Discussion

According to our clinical observations, the subjects were 2.63–4.10 days old at the beginning of the intervention and 133.77–134.43 days old at the end of the intervention. The increase in body weight, recumbent length, and head circumference was similar among the groups. According to the World Health Organization child growth standards [30], the 50th percentile for 0–4-month body weight increase is 3,636 g for boys and 3,210 g for girls, corresponding to a growth rate of 26.31–29.80 g/day. Our subjects presented a growth rate of 30.03–31.47 g/day, indicating that infant formula containing *B. animalis* CP-9 or *L. salivarius* AP-32 did not affect body weight. For 0–4-month body length increments, the 50th percentile is 14 cm for boys and 13 cm for girls, with a growth rate of 0.107–0.115 cm/day. Our subjects presented a normal growth rate of 0.104–0.107 cm/day, suggesting that the intervention did not affect body length growth. The 50th percentile for the 0–4-month head circumference increase is 7.1 cm for boys and 6.7 cm for girls, corresponding to a growth rate of 0.055–0.058 cm/day. Our subjects presented a normal growth rate of 0.059–0.062 cm/day, indicating that the growth of head circumference was not influenced by the infant formula containing *B. animalis* CP-9 or *L. salivarius* AP-32.

Unexpectedly, fewer data were analysed in the *L. salivarius* AP-32 group than anticipated, resulting in a smaller sample size than originally estimated. This reduction in sample size affected the statistical power of the study; therefore, the results should be interpreted with caution [31]. Nevertheless, interventions involving infant formula containing *B. animalis* CP-9 or *L. salivarius* AP-32 had no adverse effects on infant weight, height, or head circumference. This result was similar to that of our recent study in which these two strains were supplied at a relatively high dosage to 1-month-old infants [32]. A systematic review revealed that supplementation with certain strains may reduce the number of colic episodes, days with fever, and antibiotic use, but no significant effects on infant weight or height were observed, with a mean duration of intervention of 5.6 ± 2.8 months [33]. The present 4-month study findings suggested that the use of *B. animalis* CP-9 or* L. salivarius* AP-32 as formula additives is generally safe. However, further studies are needed to determine whether these two strains can significantly affect infant growth.

The antibiotic resistance of our probiotic strains was investigated to ensure that the material was suitable for intervention in infants. Surprisingly, *B. animalis* CP-9 did not exhibit tetracycline resistance even though it harboured four tetracycline resistance-related genes, including one *tet*(W) gene [34]. On the other hand, *B. animalis* CP-9 exhibited greater gentamicin resistance than the EFSA breakpoint for the *Bifidobacterium* genus. In general, *Bifidobacteria* species are inherently resistant to aminoglycosides due to the absence of cytochrome-mediated drug transport [35, 36]. Our gene annotation suggested that this resistance might be intrinsic, and this in silico-based screen provided a potential target for further laboratory-based investigations, such as insertional inactivation [36]. Similarly, *L. salivarius* AP-32 did not exhibit tetracycline resistance even though it harboured two drug resistance-related genes, one related to tetracycline resistance, on the larger plasmid [37, 38]. On the other hand, *L. salivarius* AP-32 displayed greater kanamycin resistance than the EFSA breakpoint value for facultative heterofermentative *Lactobacillus*. However, this resistance is not unique to the *L. salivarius* AP-32 strain [39]. Despite our gene annotation, kanamycin resistance might be intrinsic to *L. salivarius* AP-32, but further laboratory-based investigations are required to determine the underlying mechanism [40].

The dynamic evolution of the gut microbiota composition progresses rapidly during the first 6 months after birth. The relative abundances of some genera that are abundant at birth decrease, whereas the abundances of other genera begin to increase at measurable levels as infants age [41]. The subjects in this study were healthy, full-term, spontaneous vaginally delivered infants, which minimized the influence of delivery mode on the initial microbiota [42]. Notably, a more diverse microbiota was observed among the three groups before the intervention, and the microbiota composition became more uniform by the end of the intervention. For example, the proportion of Proteobacteria was slightly greater in the AP-32 group than in the other two groups on day 0, and this proportion was significantly lower by month 4. Proteobacteria include a wide variety of pathogenic genera, and an increase in the abundance of Proteobacteria in the gut microbiota can indicate a potential disruption in the microbial balance of the gut or dysbiosis [43]. Various factors, such as inflammation, metabolic disorders, gastrointestinal conditions, altered gut barrier function, risk of infection and childhood asthma, are associated with overgrowth or increased abundance of this phylum [44–46]. Therefore, the greater proportion of Proteobacteria and lower proportion of *Bifidobacterium* may indicate intestinal microflora dysbiosis in the AP-32 group before the intervention. Dysbiosis is possibly related to the slightly higher incidence of gastrointestinal and allergic events recorded in that group. Further investigations, including the use of germ-free mice or longer interventions and additional follow-up periods, are needed to determine whether microbiota modulation can lead to improvements in gastrointestinal and allergic conditions.

Infant formula serves as a human milk substitute, offering nutritional solutions for infants who cannot receive sufficient breast milk from their mothers. Although supplementation with one or a few probiotic strains cannot replicate the complexity of human milk, which contains numerous bioactive compounds, including oligosaccharides and immune cells, as well as varying levels of bacteria and their metabolites [47], infant formula can become more comprehensive, as additional safe probiotic strains are considered for use in infants.

## Conclusions

The present study demonstrated that the bacterial strains *B. animalis* CP-9 and *L. salivarius* AP-32 exhibit low resistance to antimicrobials and are associated with no adverse health effects when included as supplements in infant formula and fed to healthy infants. While supplementation with these strains did not affect normal growth or support the normal evolution of the gut microbiota, the available evidence confirms the safety of these bacterial strains for consumption in infants and validates the theoretical application of probiotics in this population. Nevertheless, further research is necessary to fully establish the long-term safety and efficacy of these strains, including investigations using germ-free mice and extended intervention periods, to provide a comprehensive understanding of their potential benefits and ensure their suitability for prolonged use.

## Supplementary Information

Supplementary Material 1.

Supplementary Material 2.

Supplementary Material 3.

Supplementary Material 4.

## Data Availability

The data supporting the findings of this study have been deposited in the BioProject with the primary ID PRJNA1051835 [http://www.ncbi.nlm.nih.gov/bioproject/1051835].
