# Supplementary material for: Assessment of the safety and gut microbiota modulation ability of an infant formula containing Bifidobacterium animalis ssp. lactis CP-9 or Lactobacillus salivarius AP-32 and the effects of the formula on infant growth outcomes: insights from a four-month clinical study in infants under two months old
Source: BMC Pediatr. 2024 Dec 27;24:840. doi: 10.1186/s12887-024-05289-7 (PMC11674581; doi:10.1186/s12887-024-05289-7)
Supplement: Supplementary file 1 — Supplementary Material 1. [file 12887_2024_5289_MOESM1_ESM.pdf]

Table S1. The drug resistance-related gene annotation of *B. animalis* CP-9. Seven drug resistance-related gene fragments were annotated, and two gene fragments were related to aminoglycoside resistance. Among these genes, only one possible promoter sequence structure was found 6 bases upstream of the aminoglycoside phosphotransferase gene with an *E*-value of 0.001967.

Number	Gene annotation	Related antibiotics	Mechanism	Location	Promoter site, E value	Sequence
1	Glycopeptide antibiotics resistance protein	Glycopeptide	antibiotic target protection	Chromosome	no obvious promoter found	—
2	Drug resistance transporter EmrB/QacA subfamily 4	Multidrug	Efflux Pump	Chromosome	no obvious promoter found	—
3	Probable Aminoglycoside 3′ -phosphotransferase (EC 2.7.1.95)	Aminoglycoside	antibiotic target protection	Chromosome	no obvious promoter found	—
4	Aminoglycoside phosphotransferase	Aminoglycoside	antibiotic target protection	Chromosome	"-6", "0.001967"	5'-AATATAGCCGATGGC GCGTCCTGTGGCAG-3'
5	Tetracycline resistance protein (TetW)	tetracycline	antibiotic target protection	Chromosome	no obvious promoter found	—
6	Ribosome protection-type tetracycline resistance related proteins	tetracycline	antibiotic target protection	Chromosome	no obvious promoter found	—
7	Similar to tetracycline resistance protein	tetracycline	antibiotic target protection	Chromosome	no obvious promoter found	—
