# Supplementary material for: Assessment of the safety and gut microbiota modulation ability of an infant formula containing Bifidobacterium animalis ssp. lactis CP-9 or Lactobacillus salivarius AP-32 and the effects of the formula on infant growth outcomes: insights from a four-month clinical study in infants under two months old
Source: BMC Pediatr. 2024 Dec 27;24:840. doi: 10.1186/s12887-024-05289-7 (PMC11674581; doi:10.1186/s12887-024-05289-7)
Supplement: Supplementary file 2 — Supplementary Material 2. [file 12887_2024_5289_MOESM2_ESM.pdf]

Table S2. The drug resistance-related gene annotation of *L. salivarius* AP-32. One aminoglycoside phosphotransferase family protein [APH (3')] was annotated on the circular chromosome, along with a possible promoter sequence structure located 37 bases upstream of the gene (*E*-value=0.000000, Table S2). On the larger plasmid, two drug resistance-related genes were annotated: one related to tetracycline resistance and one related to multidrug resistance. However, no possible promoter sequence structure was found upstream of these genes. Therefore, the increased kanamycin resistance of *L. salivarius* AP-32 is likely attributed to the presence of the aminoglycoside phosphotransferase family protein [APH (3')] gene with a potential upstream promoter.

Number	Gene annotation	Related antibiotics	Mechanism	Location	Promoter site, E value	Sequence
1	Permease of the drug/metabolite transporter (DMT) superfamily	Multidrug	Efflux Pump	Chromosome	no obvious promoter found	—
2	Multi antimicrobial extrusion protein (Na(+)/drug antiporter), MATE family of MDR efflux pumps	Multidrug	Efflux Pump	Chromosome	no obvious promoter found	—
3	Multidrug resistance protein B(MRP2 or ABCC2)	Multidrug	Efflux Pump	Chromosome	no obvious promoter found	—
4	ABC-type multidrug/protein/lipid transport system	Multidrug	Efflux Pump	Chromosome	no obvious promoter found	—
5	aminoglycoside phosphotransferase family protein(APH(3'))	Kanamycin	antibiotic target protection	Chromosome	"-37", "0.000000"	5′ -**TTGACA**CCTAATAGA TATATTTG**TAGACT**- ′3
6	Quinolone resistance protein norA	Quinolone	Efflux Pump	Chromosome	no obvious promoter found	—
7	Ribosome protection-type tetracycline resistance related protein group 2	Tetracycline	antibiotic target protection	Plasmid	no obvious promoter found	—
8	ABC-type multidrug transport system, permease component	Multidrug	Efflux Pump	Plasmid	no obvious promoter found	—
