# Supplementary material for: Assessment of the safety and gut microbiota modulation ability of an infant formula containing Bifidobacterium animalis ssp. lactis CP-9 or Lactobacillus salivarius AP-32 and the effects of the formula on infant growth outcomes: insights from a four-month clinical study in infants under two months old
Source: BMC Pediatr. 2024 Dec 27;24:840. doi: 10.1186/s12887-024-05289-7 (PMC11674581; doi:10.1186/s12887-024-05289-7)
Supplement: Supplementary file 3 — Supplementary Material 3. [file 12887_2024_5289_MOESM3_ESM.pdf]

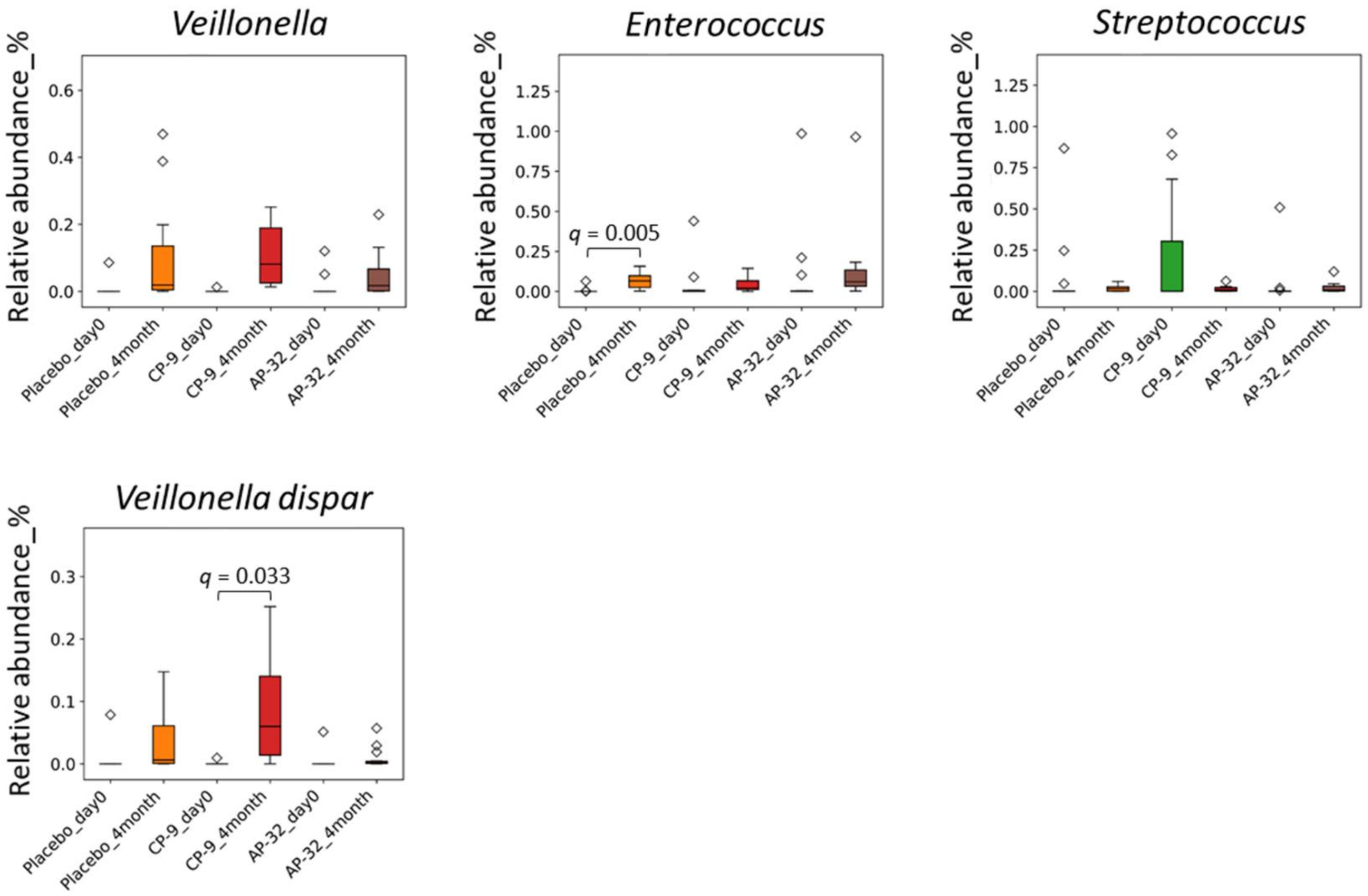

Figure S1. The genus and species involved in the modulation of the microbiota in fecal samples collected on day 0 and month 4. The *q* value was the *p* value adjusted using the FDR with the BH correction.
