# Supplementary material for: Assessment of the safety and gut microbiota modulation ability of an infant formula containing Bifidobacterium animalis ssp. lactis CP-9 or Lactobacillus salivarius AP-32 and the effects of the formula on infant growth outcomes: insights from a four-month clinical study in infants under two months old
Source: BMC Pediatr. 2024 Dec 27;24:840. doi: 10.1186/s12887-024-05289-7 (PMC11674581; doi:10.1186/s12887-024-05289-7)
Supplement: Supplementary file 4 — Supplementary Material 4. [file 12887_2024_5289_MOESM4_ESM.pdf]

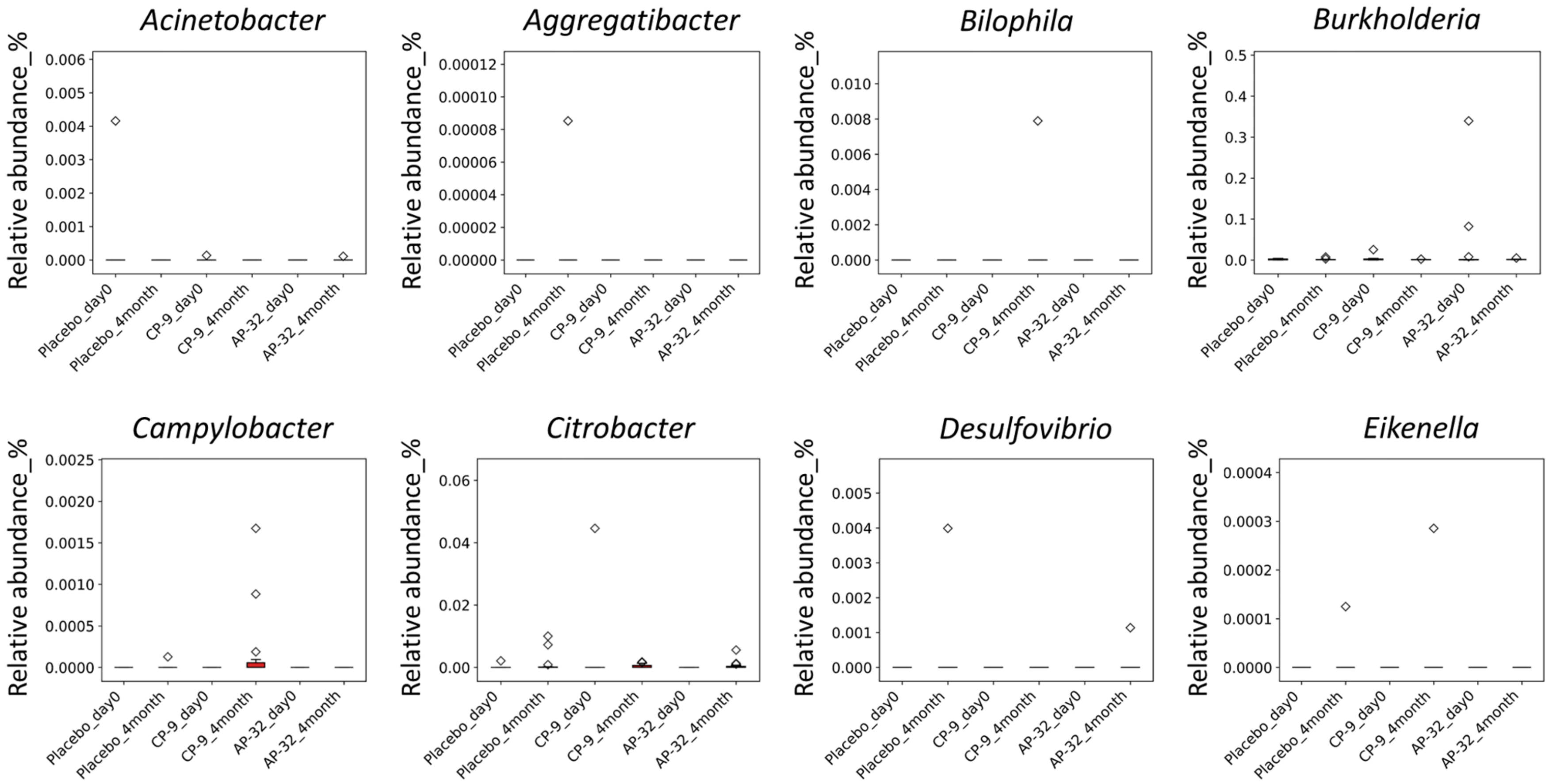

Acinetobacter
Aggregatibacter
Bilophila
Burkholderia
Campylobacter
Citrobacter
Desulfovibrio
Eikenella
Relative abundance_%
Placebo_day0
Placebo_4month
CP-9_day0
CP-9_4month
AP-32_day0
AP-32_4month

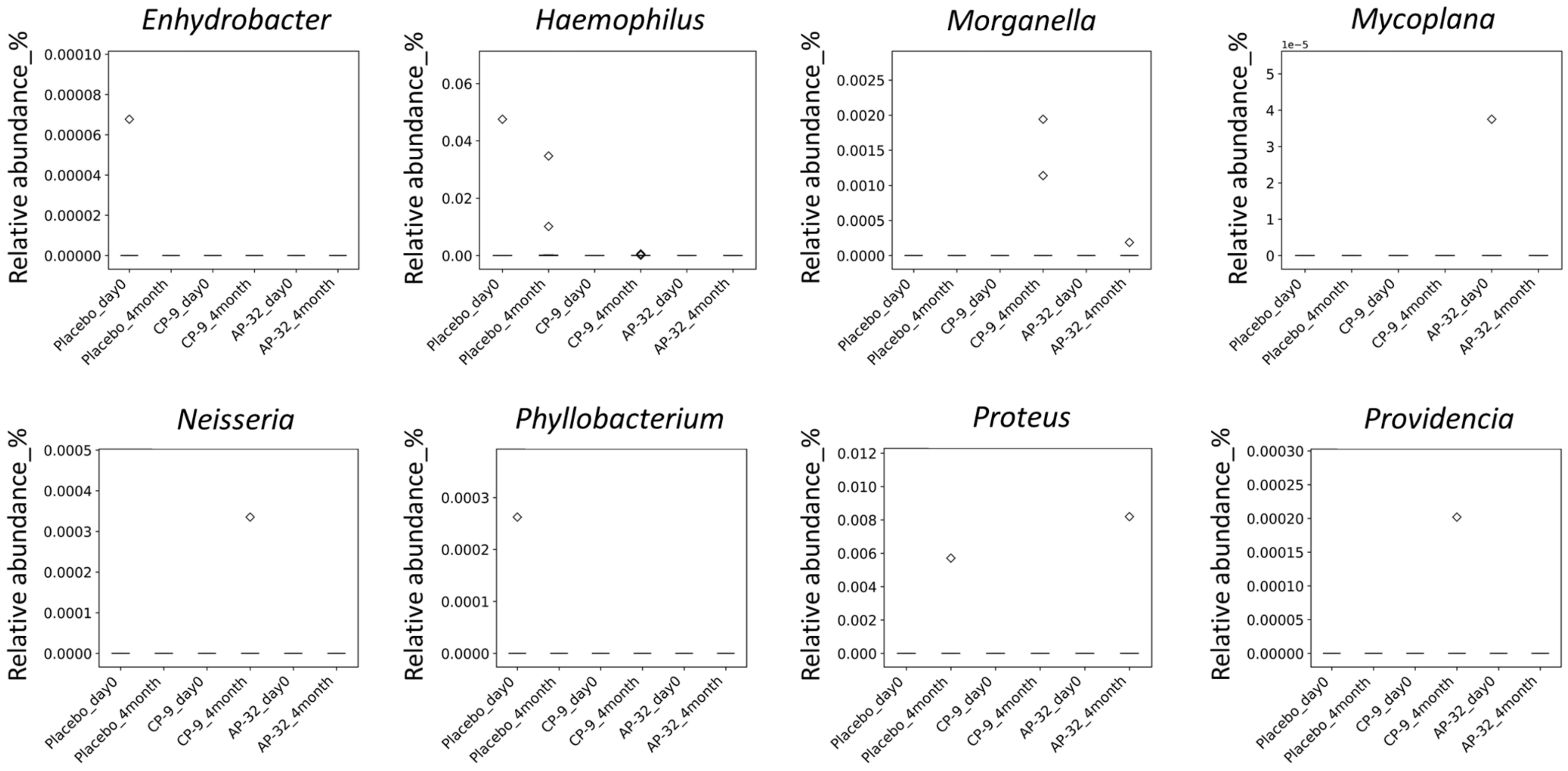
Enhydrobacter
Haemophilus
Morganella
Mycoplana
Neisseria
Phyllobacterium
Proteus
Providencia
Relative abundance_%
1e−5
Placebo_day0
Placebo_4month
CP-9_day0
CP-9_4month
AP-32_day0
AP-32_4month

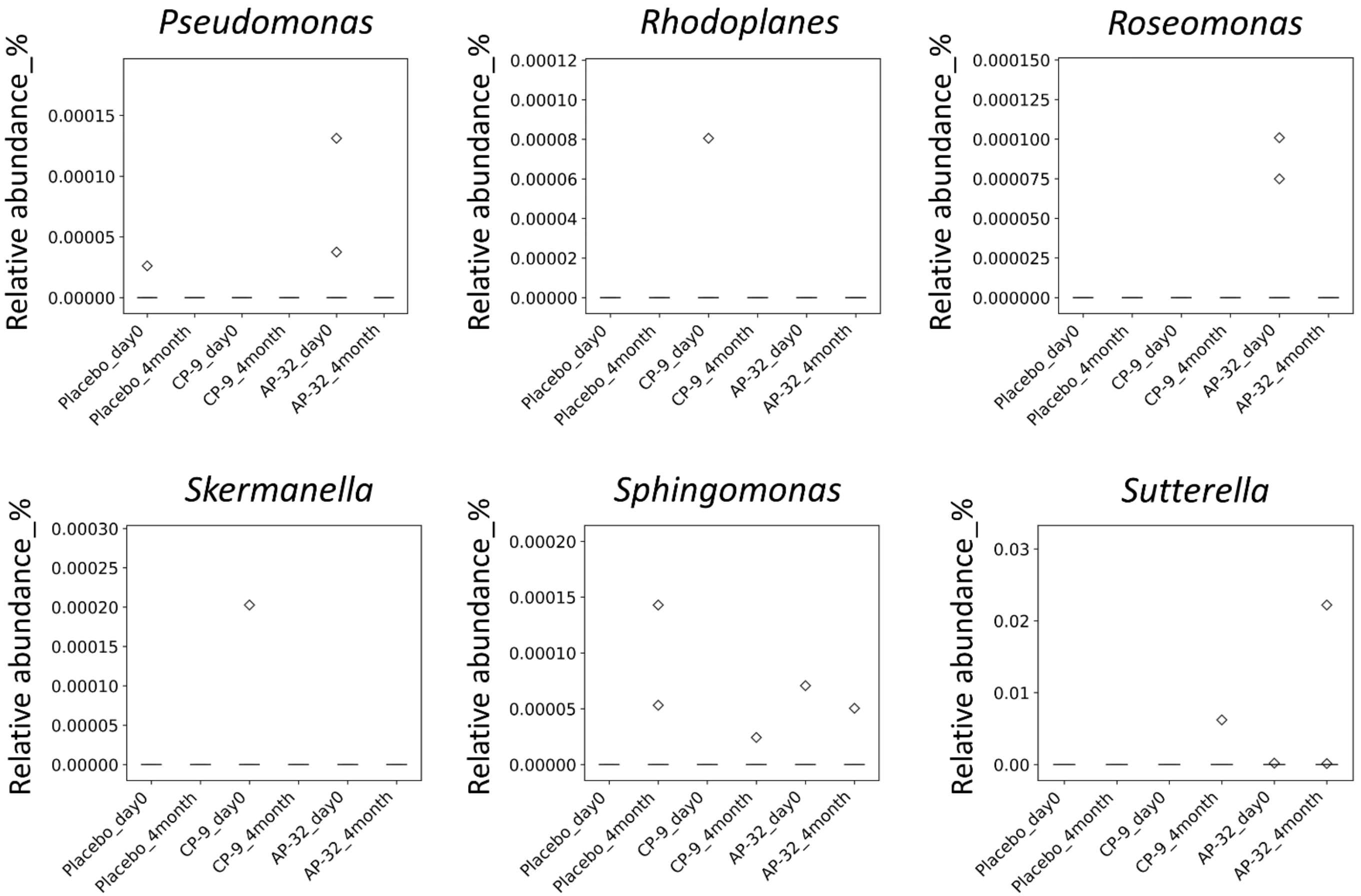

Figure S2. No significant alteration was detected in twenty-two genera of Proteobacteria phylum in the fecal samples collected on day 0 and month 4. The *q* value was the *p* value adjusted using the FDR with the BH correction.
